# Supplementary figures and images for: Using underwater video to evaluate the performance of the Fukui trap as a mitigation tool for the invasive European green crab (Carcinus maenas) in Newfoundland, Canada
Source: PeerJ. 2018 Jan 10;6:e4223. doi: 10.7717/peerj.4223 (PMC5767085; doi:10.7717/peerj.4223)

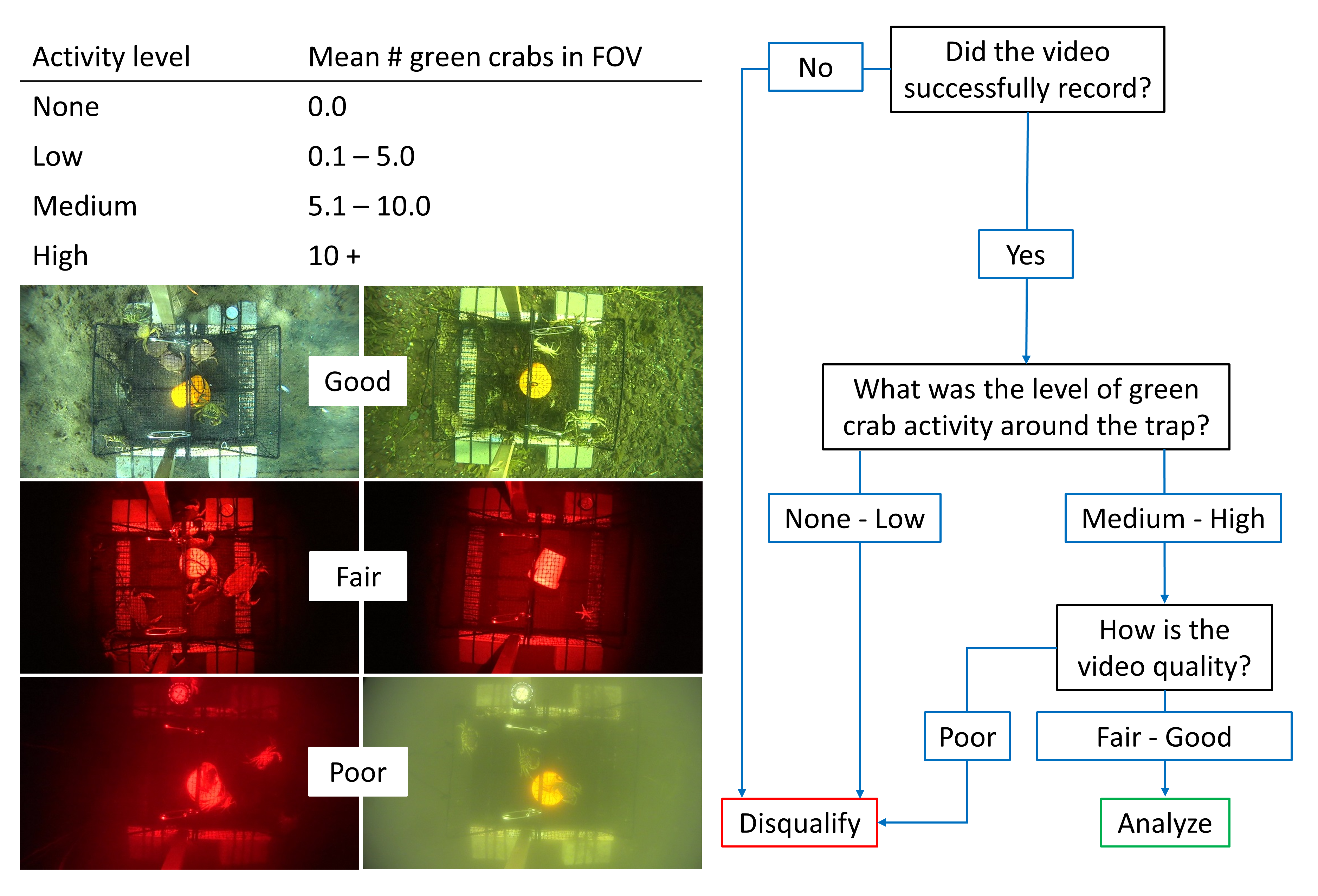

Supplement: Figure S1 [file peerj-06-4223-s001.png]
